# Emergency Medicine Resident Perceptions of Their Own Throughput Metrics

**DOI:** 10.7759/cureus.114470

**Published:** 2026-08-13

**Authors:** Christian Cochrane, Pascal Escobar, Vanessa Cardenas, Ariel Vera, Joseph Ray, David Lebowitz

**Affiliations:** 1 Emergency Medicine, University of Central Florida/HCA Florida Healthcare Graduate Medical Education (Greater Orlando/Osceola), Orlando, USA

**Keywords:** emergency medicine, medical education, residents, systems-based practice, throughput

## Abstract

Background

Operational throughput metrics, such as emergency department (ED) length of stay (LOS) and time to disposition, are frequently and routinely tracked in EDs, but they are not usually integrated into resident education as structured formative feedback. The educational impact of routinely sharing individualized metrics with emergency medicine (EM) residents remains unclear.

Methods

We conducted a prospective pre-post quality improvement (QI) study at a single academic EM residency program between May and September 2025. Post-graduate year two and three residents received weekly individualized throughput metrics, including LOS, greet-to-order time, and disposition time, derived from existing departmental data systems. Metrics were then distributed in a standardized manner for a period of three months. Residents completed structured electronic surveys before and after implementation. Primary outcomes included self-reported perceptions of educational value, workflow impact, emotional response, and behavioral changes.

Results

A total of 10 residents completed both the pre- and post-intervention surveys. At baseline, familiarity with personal throughput metrics was limited. After three months of metric exposure, 70% rated the metrics as somewhat useful or better. Half of the residents reported making minor changes to their clinical practices. Most residents (60%) reported no stress or anxiety related to receiving these metrics. Reflection on their workflow and recognition of inefficiencies increased, while documentation timeliness and mentor engagement were largely unchanged. However, interest in continued metric use was high (80%).

Discussion

Weekly individualized throughput metrics were feasible to implement and generally well tolerated by EM residents. Exposure to performance data was associated with increased self-reported reflection and modest behavioral adjustments without a significant emotional burden. Based on this, performance metrics may serve as a useful adjunct to residency education when framed as formative feedback. These findings suggest that integration with structured mentorship may be necessary to achieve sustained behavioral changes. This implementation strategy provides a framework for future studies evaluating structured mentorship or coaching alongside individualized performance metrics.

Conclusion

EM residents receiving a weekly metric report have modest behavioral adjustments regarding the metrics received. Residents reported minimal emotional burden after this strategic implementation of weekly individualized throughput metrics.

## Introduction

Timeliness and efficiency are core domains of quality in emergency medicine, with direct implications for patient safety, departmental throughput, and patient satisfaction. Prolonged emergency department (ED) length of stay (LOS) and delays in disposition have been associated with adverse outcomes, overcrowding, and decreased patient experience, making throughput metrics central to institutional quality improvement (QI) efforts [1]. In academic EDs, residents play a substantial role in patient evaluation, decision-making, and disposition, and their individual workflow patterns can meaningfully influence operational performance. Accordingly, metrics such as LOS, door-to-provider time, and time to disposition are routinely captured through electronic health records and departmental dashboards [2]. However, these data findings are most often used for operational oversight and reporting rather than intentionally incorporated into resident education.

Although EM residents routinely generate large volumes of operational performance data, these metrics are rarely shared with trainees in a consistent, structured, or educationally grounded manner. While the Accreditation Council for Graduate Medical Education (ACGME) emphasizes practice-based learning, self-assessment, and progressive autonomy [3], there is limited guidance on the routine use of individualized throughput metrics as formative feedback. As a result, a gap persists between the widespread availability of resident performance data and its intentional use to promote reflection, behavior change, and professional growth.

Existing literature on resident-facing performance metrics remains limited. Sebok-Syer et al. [4] highlighted the growing availability of electronic health record-derived metrics but noted that these data are infrequently deployed as structured educational tools. Mamtani et al. [5] found that providing resident physicians with performance metric scorecards improved resident satisfaction but did not result in measurable changes in clinical performance, underscoring the need to better understand how trainees perceive and respond to such data. In contrast, broader audit-and-feedback literature suggests that timely, individualized feedback can promote self-reflection and support deliberate practice, particularly when feedback is framed as formative rather than punitive [6,7]. There is already a push to incorporate structural and/or organizational changes to reduce burnout amongst physicians [8]. Whether these principles apply to operational throughput metrics in the ED remains unclear.

We hypothesized that providing emergency medicine residents with regular access to individualized throughput metrics would be perceived as useful and educational and would support self-reflection and workflow adjustment. The objective of this study was to evaluate residents' perceptions of receiving weekly personal throughput metrics and to determine whether access to these data was associated with self-reported reflection, workflow changes, or perceived effects on learning and well-being.

## Materials and methods

Study design

This pilot study was conducted as a quality improvement (QI), prospective, pre-post observational study evaluating emergency medicine residents' perceptions of receiving regular individualized performance metrics. The intervention consisted of providing residents with weekly throughput metrics over the study period of three months, with assessment of perceptions and self-reported behavioral impact before and after exposure. Initial surveys were delivered in May 2025, and post-intervention surveys were given in September 2025.

Setting and participants

The study was conducted within a single community/academic hybrid emergency medicine residency program. Inclusion criteria were all emergency medicine senior residents, post-graduate year (PGY) 2 or 3, during the study period. There were no formal exclusion criteria beyond non-participation in either the pre- or post-intervention survey. Because this was a QI initiative involving all eligible residents in the program, no a priori sample size calculation was performed. Ultimately, a convenience sample consisting of all eligible PGY-2 and PGY-3 residents at the study institution was used.

Intervention

Residents of post-graduate year (PGY) 2 and 3 received weekly individualized performance metrics, which included commonly tracked ED throughput measures, such as LOS, greet-to-order time, and disposition time. Metrics were derived from existing departmental data systems and distributed in a consistent, standardized format every week. Residents received anonymized data for the entire program, but were told their own personal unique identifier. This allowed all residents to see all the data, but they could only identify their own. An example of what this scorecard looks like can be found in the Appendix.

Beyond simply receiving their weekly metrics, no further feedback regarding the metrics was provided. Faculty or administration did not use these metrics during evaluations or feedback sessions unless brought up specifically by the resident. The intervention was intended to provide regular, actionable feedback to residents regarding patient flow and efficiency, without linking metrics to formal evaluation or punitive measures.

Data collection and survey instrument

Data were collected using an internally developed, structured survey administered electronically at two time points. The questionnaire was developed by the investigators and reviewed by the core teaching faculty of the residency program for face validity and clarity of reading. The questions were chosen specifically for this QI initiative and consisted of structured Likert-type and categorical response items. The questionnaire was not formally validated prior to implementation. First, prior to sending any metric data, a baseline survey was conducted. Then, after three months of sharing the metrics, a post-intervention survey was completed (Table 1).

**Table 1:** Pre-intervention and post-intervention survey questions

Pre-survey Questions	Post-survey Questions
How familiar are you with your own personal throughput metrics (e.g., Length of stay (LOS), greet-to-order, disposition time)?	How useful were the weekly performance metrics in helping you reflect on or improve your clinical workflow?
How useful do you think individual metrics (e.g., LOS by emergency severity index (ESI), greet-to-order) are for improving clinical practice?	Did reviewing your metrics lead you to change or consider changing any aspect of your clinical practice?
Do you feel that reviewing your own throughput metrics would motivate you to change your behavior or clinical workflow?	Did receiving the metrics cause any stress, anxiety, or other emotional impact?
Do you anticipate any negative emotional impact (e.g., stress, anxiety) from receiving this data regularly?	Did you discuss your performance metrics with a mentor attending?
How often do you consciously adjust your workflow to improve throughput?	How often do you consciously adjust your workflow to improve throughput after receiving metrics?
How frequently do you reflect on your patient flow or time-to-disposition after a shift?	How often did you reflect on your patient flow or time to disposition after receiving metrics?
How comfortable are you discussing your throughput metrics with a faculty mentor or attending?	Did the metrics help you better recognize inefficiencies or bottlenecks in your workflow?
Do you believe the time from the last result to disposition is something you can actively control during a shift?	How confident do you feel now in your ability to manage multiple patients efficiently (task-switching)?
Do you feel confident in your ability to recognize bottlenecks in your own workflow (e.g., delay in placing disposition orders)?	Did the metrics impact how timely you completed documentation and sign notes?
Do you usually complete your documentation and sign notes within 24 hours of a shift?	How likely are you to continue making use of these metrics moving forward?
Would reviewing anonymized peer comparisons motivate you to improve your workflow?	Would you like to continue receiving weekly performance metrics?
	Overall, how satisfied were you with the way the metrics were shared and explained?
	To what extent did the metrics help you grow as a physician?

Study variables and analysis

Residents who completed both surveys were included in the analysis. Data were analyzed using descriptive statistics. Categorical variables are presented as frequencies and percentages. Because this was an exploratory QI project with a small sample size and primarily descriptive objectives, inferential statistical testing was not performed. Instead, emphasis was placed on observed response distributions and absolute differences between pre- and post-intervention survey responses.

Ethical considerations

This project was conducted as a QI initiative using existing institutional data. Participation in surveys was voluntary, and responses were de-identified prior to analysis. Survey completion was voluntary, and responses were not linked to individuals. Completion of the survey implied consent. The study was conducted in accordance with institutional policies governing QI activities. It was deemed IRB exempt from institutional oversight.

## Results

A total of 10 emergency medicine residents completed both the pre-intervention and post-intervention surveys. Respondents included six rising PGY-2 residents (60%) and four rising PGY-3 residents (40%). Baseline survey results are detailed in Table 2, and post-intervention survey results are in Table 3.

**Table 2:** Baseline resident perceptions of weekly throughput metrics (N = 10)

Survey Item	Response Category	n (%)
Familiarity with personal throughput metrics	Not at all familiar	4 (40)
	Slightly familiar	6 (60)
Perceived usefulness of individual metrics	Not useful	1 (10)
	Somewhat useful	4 (40)
	Useful	4 (40)
	Very useful	1 (10)
Anticipated motivation to change workflow	Not at all	1 (10)
	A little	1 (10)
	Somewhat	5 (50)
	Very much so	2 (20)
Anticipated negative emotional impact	None	0 (0)
	A little	6 (60)
	Somewhat	4 (40)
Frequency of workflow adjustment to improve throughput	Never	0 (0)
	Sometimes	2 (20)
	Often	6 (60)
	Always	2 (20)
Frequency of post-shift reflection on patient flow	Never	2 (20)
	Occasionally	3 (30)
	Frequently	3 (30)
	After every shift	2 (20)
Comfort discussing metrics with faculty	Not at all	0 (0)
	Somewhat comfortable	5 (50)
	Comfortable	0 (0)
	Very comfortable	5 (50)
Perceived control over time from last result to disposition	No	1 (10)
	Sometimes	7 (70)
	Often	1 (10)
	Yes	1 (10)
Confidence recognizing workflow bottlenecks	Not at all	0 (0)
	Somewhat confident	7 (70)
	Confident	3 (30)
	Very confident	0 (0)
Documentation completed within 24 hours	Never	0 (0)
	Sometimes	4 (40)
	Usually	3 (30)
	Always	3 (30)
Motivation from anonymized peer comparison	No	1 (10)
	Maybe	2 (20)
	Probably	5 (50)
	Definitely	2 (20)

**Table 3:** Post-intervention resident responses to receiving weekly throughput metrics (N = 10)

Survey Item	Response Category	n (%)
Usefulness of weekly performance metrics	Not useful	3 (30)
	Somewhat useful	4 (40)
	Useful	1 (10)
	Very useful	2 (20)
Metrics prompted change or consideration of change in clinical practice	No	3 (30)
	Somewhat	2 (20)
	Yes, minor changes	5 (50)
Emotional impact from receiving metrics	Not at all	6 (60)
	A little	3 (30)
	Somewhat	1 (10)
Discussion of metrics with a faculty mentor	No	9 (90)
	Yes, and it was helpful	1 (10)
Frequency of workflow adjustment after receiving metrics	Never	3 (30)
	Sometimes	3 (30)
	Often	4 (40)
Frequency of post-shift reflection on patient flow	Never	1 (10)
	Occasionally	5 (50)
	Frequently	4 (40)
Recognition of inefficiencies or workflow bottlenecks	Not at all	2 (20)
	Somewhat	5 (50)
	Yes	3 (30)
Confidence managing multiple patients (task-switching)	Somewhat confident	2 (20)
	Confident	7 (70)
	Very confident	1 (10)
Impact on documentation timeliness	No impact	7 (70)
	Somewhat	3 (30)
Likelihood of continued use of metrics	Not likely	1 (10)
	Somewhat likely	5 (50)
	Likely	3 (30)
	Very likely	1 (10)
Desire to continue receiving weekly metrics	Yes	8 (80)
	No	2 (20)
Satisfaction with delivery and explanation of metrics	Not satisfied	1 (10)
	Somewhat satisfied	2 (20)
	Satisfied	7 (70)
Perceived professional growth from metrics	Not at all	2 (20)
	A little	4 (40)
	Somewhat	4 (40)

Prior to providing residents with their weekly performance metrics, their familiarity with throughput metrics was limited. However, most residents anticipated motivation from anonymized peer comparison. Regarding their perceived ability to influence throughput, most residents (70%) felt they only sometimes had control over the time from last result to disposition.

Overall, residents reported generally favorable perceptions of individualized throughput metrics following the intervention. While perceptions of the usefulness of the metrics became more variable after three months of exposure, half of the residents reported making minor changes to their clinical practice after reviewing their performance data, and many continued to feel they were consciously adjusting their workflow to improve throughput.

Residents also reported continued awareness of workflow bottlenecks after receiving individualized metrics, although documentation timeliness was largely unchanged. Despite high baseline comfort discussing performance metrics with faculty, few residents reported actually discussing their individualized metrics with a faculty mentor during the study period.

The intervention was associated with minimal reported emotional burden. Most residents reported no stress or anxiety related to receiving individualized performance metrics, while reflection on patient flow remained common through the intervention.

Most residents reported at least some perception of growth as a physician after receiving individualized throughput metrics, although relatively few described substantial growth. Confidence in managing multiple patients remained high, and most residents reported being satisfied with the method used to distribute the metrics. Although many residents indicated they were only somewhat likely to continue using the metrics independently, 80% expressed a desire to continue receiving weekly metric reports.

## Discussion

In this pilot QI study, emergency medicine residents generally perceived individualized throughput metrics as acceptable and educationally valuable, with little reported emotional burden. Per the ACGME common program requirements, "Residents and faculty members must receive data on quality metrics and benchmarks related to their patient populations" [9]. Providing this level of data to residents helps meet this important ACGME requirement while potentially preparing resident physicians for the metrics they will be measured on as attending physicians.

Initially, residents reported limited familiarity with their own throughput metrics despite anticipating that such data could be motivating. This finding aligns with prior work demonstrating that operational performance data is widely generated in academic emergency departments but is infrequently shared with residents in a structured manner [4].

One concern about deploying this level of metric data to residents was the potential negative impact on resident emotions, particularly if they were below their peers in various metrics. The data demonstrated that emergency medicine residents generally viewed weekly individualized throughput metrics as acceptable and educationally useful, with limited reported emotional burden. From a system-based practice perspective, one of the goals of sharing metric data was to motivate residents to develop awareness of potential inefficiencies in ED throughput and work to address this on shift. Following their exposure to weekly metrics, residents had modest self-reported practice changes, consistent with prior studies showing that the performance scorecards can improve satisfaction and awareness without reliably producing immediate or large changes in measurable performance [5].

Despite general acceptance of the metrics, engagement with faculty mentors around performance data was limited, as 90% had no formal discussion with a faculty member regarding their metrics. One theory may suggest that feedback alone is not sufficient to drive sustained learning without opportunities for dialogue and guided reflection [10,11]. Without faculty or mentors specifically discussing the metrics, it is easily possible that residents do not engage with them, or they do initially but lose interest as they become simply another rote e-mail they receive. Further research into the integration of metrics into structured mentorship or coaching frameworks and their effects on educational impact or emotional burden is needed.

Throughput metrics are often a difficult subject to discuss due to the negative perception surrounding prioritizing them while actively on shift, caring for patients [12]. Most respondents were at least satisfied (70%) or somewhat satisfied (20%) with the approach chosen to deliver and explain the metrics. The strategy used in this study to distribute data was carefully chosen to minimize emotional burden from metrics. Residents can see their own numbers, but also what the general values are for the other residents. However, they cannot compare themselves to any specific peer, as this was anonymized. The intention with this method was to encourage self-reflection while discouraging judgment towards others. We believe this approach to distribution played a large role in the relatively positive, or at least not negative, perception of receiving their metrics.

Some limitations of this study are the single institution design, small sample size, reliance on self-report to the outcomes, and the absence of objective throughput performance measures. The study evaluated residents' perceptions and self-reported behavioral responses rather than objective changes in throughput performance and therefore cannot determine whether exposure to metrics directly improved operational efficiency. Similarly, despite the anonymity emphasized throughout the survey and metric distribution, the self-reported nature of the survey creates a risk for response bias. Nonetheless, these findings do suggest that weekly individualized throughput metrics are feasible, acceptable to residents, and may support reflection without substantial negative emotional impact when provided in this manner. Further work and studies should explore pairing metrics with structured coaching to promote deeper engagement and sustainable Improvement.

## Conclusions

In this single-center QI pilot study, conducted within a community-based emergency medicine residency program, weekly individualized throughput metrics were feasible to implement and were generally well tolerated by emergency medicine residents. Exposure to individualized throughput data, including metrics such as ED LOS, greet-to-order time, and disposition time, was associated with minor self-reported changes in clinical practice and minimal emotional burden.

## Figures and Tables

**Figure 1:**
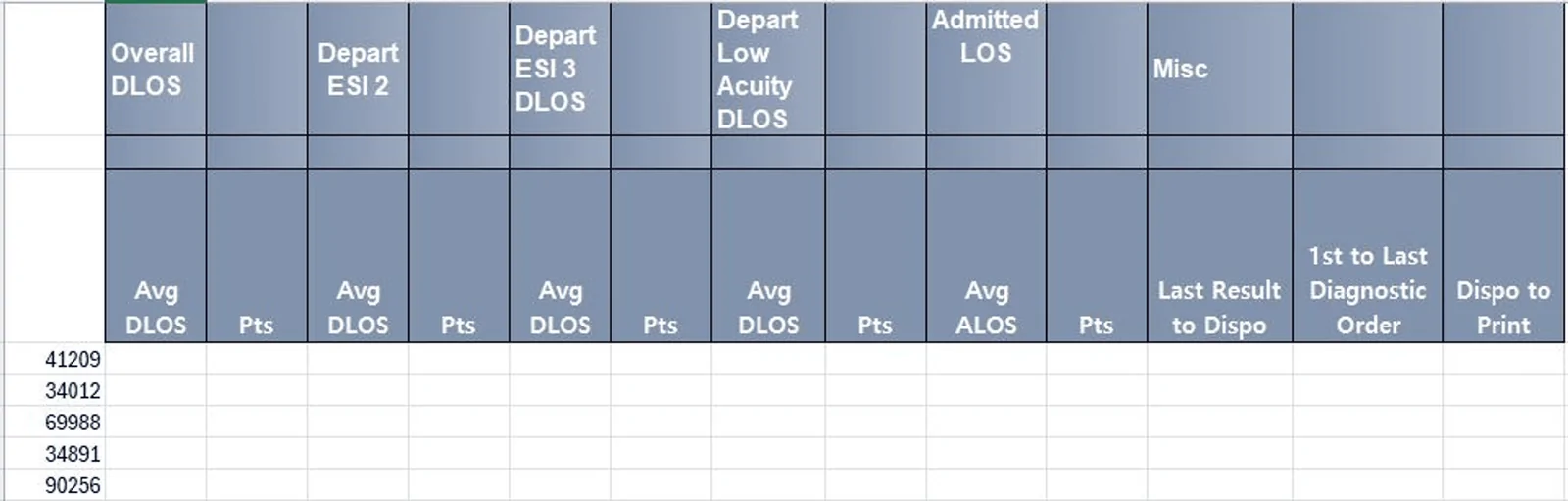
Example of the weekly scorecard provided to the senior residents The fields were intentionally left blank for publication, and the identification numbers were modified from the residents' actual identification numbers for confidentiality reasons.
